# Relating circulating thyroid hormone concentrations to serum interleukins-6 and -10 in association with non-thyroidal illnesses including chronic renal insufficiency

**DOI:** 10.1186/1472-6823-8-1

**Published:** 2008-01-22

**Authors:** Hamdy A Abo-Zenah, Sabry A Shoeb, Alaa A Sabry, Hesham A Ismail

**Affiliations:** 1 Departments Of Internal Medicine, Faculty Of Medicine, Menufiya University, Egypt; 2 Molecular Diagnostic Department, Genetic Engineering and Biotechnology Research Institute, Menufiya University, Egypt; 3 Mansura Urology and Nephrology Institute, Mansura University, Egypt

## Abstract

**Background:**

Because of the possible role of cytokines including interleukins (IL) in systemic non-thyroidal illnesses' (NTI) pathogenesis and consequently the frequently associated alterations in thyroid hormone (TH) concentrations constituting the euthyroid sick syndrome (ESS), we aimed in this research to elucidate the possible relation between IL-6 & IL-10 and any documented ESS in a cohort of patients with NTI.

**Methods:**

Sixty patients and twenty healthy volunteers were recruited. The patients were subdivided into three subgroups depending on their underlying NTI and included 20 patients with chronic renal insufficiency (CRI), congestive heart failure (CHF), and ICU patients with myocardial infarction (MI). Determination of the circulating serum levels of IL-6 and IL-10, thyroid stimulating hormone (TSH), as well as total T4 and T3 was carried out.

**Results:**

In the whole group of patients, we detected a significantly lower T3 and T4 levels compared to control subjects (0.938 ± 0.477 vs 1.345 ± 0.44 nmol/L, p = 0.001 and 47.9 ± 28.41 vs 108 ± 19.49 nmol/L, p < 0.0001 respectively) while the TSH level was normal (1.08+0.518 μIU/L). Further, IL-6 was substantially higher above controls' levels (105.18 ± 72.01 vs 3.35 ± 1.18 ng/L, p < 0.00001) and correlated negatively with both T3 and T4 (r = -0.620, p < 0.0001 & -0.267, p < 0.001, respectively). Similarly was IL-10 level (74.13 ± 52.99 vs 2.64 ± 0.92 ng/ml, p < 0.00001) that correlated negatively with T3 (r = -0.512, p < 0.0001) but not T4. Interestingly, both interleukins correlated positively (r = 0.770, p = <0.001). Moreover, IL-6 (R^2 ^= 0.338, p = 0.001) and not IL-10 was a predictor of low T3 levels with only a borderline significance for T4 (R^2 ^= 0.082, p = 0.071).

By subgroup analysis, the proportion of patients with subnormal T3, T4, and TSH levels was highest in the MI patients (70%, 70%, and 72%, respectively) who displayed the greatest IL-6 and IL-10 concentrations (192.5 ± 45.1 ng/L & 122.95 ± 46.1 ng/L, respectively) compared with CHF (82.95 ± 28.9 ng/L & 69.05 ± 44.0 ng/L, respectively) and CRI patients (40.05 ± 28.9 ng/L & 30.4 ± 10.6 ng/L, respectively). Surprisingly, CRI patients showed the least disturbance in IL-6 and IL-10 despite the lower levels of T3, T4, and TSH in a higher proportion of them compared to CHF patients (40%, 45%, & 26% vs 35%, 25%, & 18%, respectively).

**Conclusion:**

the high prevalence of ESS we detected in NTI including CRI may be linked to IL-6 and IL-10 alterations. Further, perturbation of IL-6 and not IL-10 might be involved in ESS pathogenesis although it is not the only key player as suggested by our findings in CRI.

## Background

Despite absence of thyroid disease, patients with non-thyroidal illness (NTI) frequently have changes in serum thyroid hormone (TH) measurements that may suggest thyroid dysfunction. The clinical impression of euthyroidism is supported by normal serum thyroid stimulating hormone (TSH) in most of these patients [1]. Many of the clinically euthyroid patients with NTI have low circulating concentrations of total and absolute free triiodothyronine (T3), low-normal concentrations of total thyroxine (T4), elevated concentrations of absolute free T4, and normal or subnormal TSH [2] although Hesch (1981) has reported simultaneous elevation of TSH to compensate for these low levels. Consequently, the patients are usually clinically euthyroid [3]. This was named the "euthyroid sick syndrome" (ESS) [4] that have been described some 36 years ago [5].

The mechanisms accounting for such alterations in the TH levels in association with NTI remain unknown despite extensive investigations. The hope for the discovery of factor (s) responsible for such changes came with the observations that inflammatory cytokines are influential in systemic diseases mediation [6]. More precisely, the cytokine tumor necrosis factor-α (TNF-α) and interleukin-1β (IL-1β) resulted in similar changes in TH concentrations in experimental animals [7] and human volunteers [8]. However, none of the two cytokines was consistently detectable NTI-patients [9,10]. In contrast, IL-6 is usually detectable in serum during illness and acts as a systemic hormone [11] that may mediate the well documented inhibitory effect of IL-1 on thyroid cell functions [12]. Interestingly, Shalaby and colleagues (1989) suggested IL-6 as a potential factor in the pathogenesis of the ESS [13].

Although the pathogenesis of hypothalamic-pituitary-thyroid axis depression encountered in ESS remains elusive, yet it is currently agreed that it may be related to increased cytokines production [14,11,15] that induced competition for limiting amounts of co-activators and decreases hepatocyte thyroxine 5' D-I deiodinase enzyme expression [16]. The inhibitory effect of IL-6 on thyroid function may be through binding of IL-6-sIL-6R complex to gp130 [17].

In contrast to IL-6, IL-10 is one of the most potent anti-inflammatory cytokines [18,19] and is produced by macrophages as well as other cell types [20]. Interestingly, pro-inflammatory stimuli like IL-1β and TNF-α enhance its secretion without any influence of IL-6 [21].

We have carried out this cross-sectional observational study to link thyroid function and the cytokines; IL-6 as well as IL-10 in a group of patients with ESS associated with variable NTI including chronic renal insufficiency (CRI).

## Methods

We have investigated serum samples collected from 60 patients (46 men, 14 women; aged 45 ± 19 years) who were hospitalized because of a wide variety of NTI. The patients were recruited to the planned study consecutively in a random manner except those with known or clinically suspected thyroid dysfunction. Additional exclusion criteria were use of thyroid hormones or thyrostatic medications. The patients were subdivided into 3 equal subgroups reflecting the nature of their NTI and include; those with CRI, congestive heart failure (CHF), and patients with acute myocardial infarction (MI). None of the CRI patient was replaced by dialysis treatment as their mean estimated glomerular filtration rate, using the abbreviated 4 variable MDRD formula, was 27.6 ± 3 ml/min/1.73 m^2^. The leading cause of their chronic renal insufficiency was DM (30%), hypertension (25%), glomerular disease (15%), obstructive nephropathy (10%) and unknown in the remaining (20%). The severity of MI was assessed clinically as well as by the degree of elevation of CK-MB isoenzyme. Twenty healthy volunteers (15 men, 5 women; aged 39 ± 5 years) were recruited as control group. Samples from both patients and controls were collected for measurement of serum IL-6, IL-10, TSH, T3, and total T4 levels.

Both, the ultrasensitive human TSH (hTSH II) and free T3 were measured by a Microparticle Enzyme Immunoassay (MEIA) on AxSYM System (Abbott Laboratories, Abbott Park, USA) [22,23] while total serum T4 was measured by the Fluorescence Polarization Immunoassay (FPIA) method on AxSYM System [24] using the standard laboratory methodologies.

Serum IL-6 was measured using a commercially obtained immunoassay (IL-6 Quantikin assay, R&D Systems, Abingdon, UK) with a sensitivity of 0.7 ng/l and an intra- and inter-assay CV of 3.2 and 5.7%, respectively. IL-6 reference values in fresh samples of healthy individuals are 20-12.5 ng/L. The serum level of IL-10 (Human IL-10 Quantikin ELISA immunoassay, R&D Systems Inc, Minneapolis, Minnesota, USA) was measured with an expected value below 5 pg/ml and no cross-reactivity with other interleukins including IL-6. The maximum inter- and intra-assay CV were 9.8% and 5.6%, respectively. Blood samples were taken between 7 AM and 9 AM after an overnight fast. After centrifugation (1500 × g) for 10 minutes, aliquots of serum were stored at -20°C until the time of sample analysis. Both interleukins were assessed by competitive enzyme-linked immunosorbent assay (ELISA) in serum of both patients' and control's according to a method described by Helle and colleagues (1991) using recombinant human cytokine as standard [25].

For the MI patients, the cardiac enzyme CK-MB isoenzyme was measured (IU/L) as a rough indicator of myocardial damage severity while the glomerular filtration rate was estimated (eGFR) based on the abbreviated MDRD formula:

eGFR (ml/min/1.73 m^2^) = 186 × {[sCr]^-1.154^} × age (years)^-0.203 ^× 0.742 if Female × 1.21 if Black.

Where: sCr = serum creatinine concentration (mg/dl).

This study was approved by our local ethical committee and all the patients participated in the study after a written consent.

### Statistical Analysis

Variables are given as mean and standard deviation (SD) unless otherwise stated. T-test, Kruskal-Wallis Test and Pearson correlation were used as indicated. Multivariate linear regression analysis was employed to determine the predictive value of quantitative parameters. A p value ≤ 0.05 was viewed as statistically significant with assignment of a borderline of significance when the p value lies between >0.05 and <0.1, while a non-significant level was noted as NS. All analysis was performed using the Statistical Package for Social Science (SPSS) version 10.0.

## Results

### Thyroid hormone levels

We noted, as seen in table 1, a lower T3 levels in patients with NTI compared to the corresponding level in control subjects (0.938 ± 0.477 vs 1.345 ± 0.44 nmol/L) that was a statistically significant (p = 0.001). Likewise was T4 concentrations (47.9 ± 28.41 vs 108 ± 19.49 nmol/L, p < 0.0001). While TSH was significantly lower in NTI patients (1.08 ± 0.518 vs 1.92 ± 0.93 μIU/L, *t *test p < 0.001), it was well within the normal reference values of employed immunoassay (0.49+4.67 μIU/L).

**Table 1:** Comparison between serum T3, T4, TSH, IL-6 and IL-10 levels in the 60 non-thyroidal illness patients and their 20 control subjects.

**Tested Parameters**	**Non-thyroidal Illness **(n = 60)	**Healthy Controls **(n = 20)	**Significance Level **(p Value)
**T3 **(n mol/L)	0.936 ± 0.47	1.343 ± 0.45	0.001
**T4 **(n mol/L)	74.90 ± 28.41	108.30 ± 14.50	<0.0001
**TSH **(μIU/L)	1.078 ± 0.52	1.921 ± 0.93	<0.001
**IL-6 **(ng/L)	105.18 ± 72.01	3.34 ± 1018	<0.0001
**IL-10 **(ng/L)	74.33 ± 52.99	2.64 ± 0.92	<0.00001

Out of the investigated 60 patients, four showed a TSH serum level that was below the sensitivity of the used assay (0.06 μIU/L). These patients were assumed to have a TSH concentration of (0.06 μIU/L) in order to calculate the mean TSH value.

### Serum interleukins concentrations

In the same table, IL-6 in NTI was significantly above controls' level (105.18 ± 72.01 vs 3.35 ± 1.18 ng/L, p < 0.00001). Overall, this level is considerably above the sensitivity of the used immunoassay kit and above, as well, the upper limit of normal reference value (<20 pg/ml). Similarly, IL-10 was significantly higher above measurements observed in controls (74.13 ± 52.99 vs 2.64 ± 0.92 ng/ml, p < 0.00001). Of note, such concentrations are significantly above the upper limit of normal level of the used kit (<5 pg/ml).

Of note, in order to verify the validity of the data in view of the high level of the investigated interleukins and to assess the linearity of the assay, we diluted the samples with the appropriate Calibrator Diluent to produce samples with values within the dynamic range of the used immunoassay.

### Relation between thyroid hormones and interleukins changes

**Figure 1:**
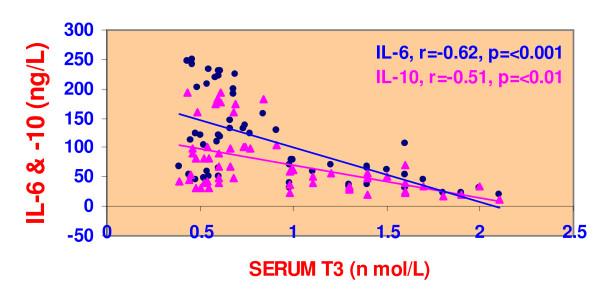
Relations between serum T3 and both IL-6 & IL-10 in all patients with non-thyroidal illness.

**Figure 2:**
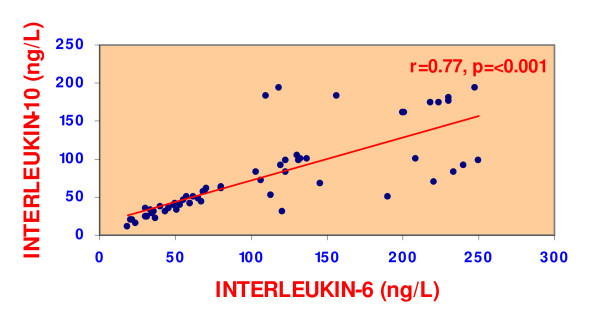
Correlation between IL-6 & IL-10 in the 60 patients with non-thyroidal illness.

We observed in NTI patients a significantly negative correlation, using Pearson' correlation coefficient, between IL-6 and T3 (r = -0.620, p < 0.0001) (figure 1). Similarly was the correlation existing between IL-6 and total T4 level (r = -0.267, p < 0.001). Likewise, IL-10 was negatively and significantly correlated with the lowered T3 in NTI-patients (r = -0.512, <0.0001) (figure 1) and not with T4 or TSH. Interestingly, both interleukins; IL-6 and IL-10 were positively and significantly correlated (r = 0.770, p = <0.001) with each other (figure 2).

By linear regression analysis, IL-6 (Unstandardized Coefficient (B) ± Standard Error (SE) = -3.72E-03 ± 0.001, R^2 ^= 0.338, p = 0.001) and not IL-10 (B) ± SE = -7.512E-04 ± 0.001, R^2 ^= 0.338, p = 0.631) was found to be a potential risk factor that could predict the observed lower circulating level of T3. However, only a borderline degree of significance (B ± SE = -0.144 ± 0.079, R^2 ^= 0.082, p = 0.071) was seen for IL-6 as a potential predictor of T4 reduction in NTI. Importantly, IL-10 has not been found to be a potential predictor for the lower TH concentrations.

### Subgroup analysis

Using ANOVA single-factor test (Kruskall-Wallis test), we have detected a significant trend in T3 alterations among the three subgroups of NTI patients and their controls (p < 0.0001). Approximately, more or less similar level of significance do exist for total T4 (p < 0.001). Interestingly, differences in both IL-6 and IL-10 among the 4 subgroups were significant also (p < 0.00001).

Further, the proportion of patients with T3, total T4, and TSH levels below normal range was highest in the MI patients (70%, 70%, and 72%, respectively) who displayed the greatest mean concentration of both IL-6 and IL-10 (192.5 ± 45.1 ng/L & 122.95 ± 46.1 ng/L, respectively) compared with CHF (82.95 ± 28.9 ng/L & 69.05 ± 44.0 ng/L, respectively) and CRI patients (40.05 ± 28.9 ng/L & 30.4 ± 10.6 ng/L, respectively) (table 2). Of note, the changes in interleukins levels matched the severity of myocardial damage in MI subgroup of patients as inferred from the significant correlations observed between the cardiac enzyme CK-MB isoenzyme and both IL-6 (r = 0.498 and p = 0.025) and IL-10 (r = 0.467 and p = 0.038). Surprising was our observation that CRI-patients showed the least disturbance in IL-6 and IL-10 despite the exhibition of lower than normal levels of T3, T4, and TSH in a higher proportion of them compared with CHF patients (40%, 45%, and 26% versus 35%, 25%, and 18%, respectively) (table 2).

**Table 2:** Prevalence and concentrations of serum T3, T4, TSH, IL-6 and IL-10 in the evaluated non-thyroidal illness subgroups of patients.

**VARIABLES**	**MI **(n = 20)	**CHF **(n = 20)	**CRI **(n = 20)
**T3 **(n mol/L)	0.63 ± 0.13 (70%)	1.07 ± 0.47 (35%)	1.14 ± 0.57 (40%)
**T4 **(n mol/L)	75.65 ± 37.57 (70%)	70.85 ± 22.68 (25%)	78.2 ± 23.47 (45%)
**TSH **(μIU/L)	1.23 ± 0.63 (72%)	0.96 ± 0.32 (18%)	1.04 ± 0.54 (26%)
**IL-6 **(ng/L)	192.55 ± 15.12	82.95 ± 18.90	40.50 ± 14.38
**IL-10 **(ng/L)	122.95 ± 46.06	69.05 ± 44.04	30.4 ± 10.57

Values in parentheses represent the percentages of patients with lower than normal concentrations of the corresponding parameter.

MI = myocardial infarction, CHF=congestive heart failure and CRI =chronic renal insufficiency.

## Discussion

In the current study, we observed a considerably lower T3 and total T4 concentrations signifying thyroid dysfunction in patients with variable non-thyroidal illnesses (NTI) while serum TSH showed a mean value that was not significantly different from that in controls. Our findings are in concordance with that noted by Horimoto and co-workers (1988) [26]and in contrast to the result of a study undertaken by Kayima and associates (1992) [27]. The clinically euthyroid patients, are biochemically abnormal defining the previously described euthyroid sick syndrome (ESS) [4,28].

Speculations as to the value of ESS development in patients with NTI have long been heard. Some investigators reported a protective function of this phenomenon [4,14] while others viewed it either as an adaptive response to reduce tissue energy requirements in face of systemic illness, or a maladaptive one, that induces damaging tissue hypothyroid [29].

Also, in the study under discussion we detected a substantially high level of the pro-inflammatory cytokine, IL-6 in NTI patients supporting its possible role as an endocrine cytokine with a regulatory effect on many endocrine systems [30], including thyroid gland [31]. We as well detected a considerably high level of the anti-inflammatory cytokine, IL-10 in NTI-patients. Likewise, Dehoux and associates (2000) reported its release in response to stressful situations such as cardiopulmonary bypass [32]. Interestingly, we noticed a positive association between both IL-6 and IL-10 which accords with a previous notion [33] and could be attributed to the fact that secretion of both interleukins is stimulated by the same cytokines such as TNF-α [34,35]. So, within the cytokine network, activation of pro-inflammatory mediators such as IL-6 is followed by increased production of endogenous inhibitory molecules including the antagonistic cytokine IL-10 in an attempt to suppress release of pro-inflammatory cytokines. This dimorphic response may be related to macrophages resistance to the suppressive effect of IL-10 as a result of down-regulation of soluble IL-10 receptors expression [36]. The high IL-10 levels was hoped for to minimize the deleterious effect of the raised IL-6 [37]. Taniguchi and colleagues (1999) highlighted this potential protective effect of IL-10 in their 25 patients with systemic inflammatory states [38].

In this study, the suppressed THs were negatively associated with IL-6 elevations. Boelen and colleagues (1993) have observed similar correlation in their 100 patients with NTI during their first day of hospitalization [11]. However, such correlation does not exist for TSH and IL-6 and this was not surprising since TSH was maintained within the normal reference value. Also, we observed an inverse association between the high IL-10 and the suppressed TH levels, in contrast to the findings of Guillén and associates [39]. Dissimilar to IL-6, IL-10 was not indicative of the observed TH alterations. Our results do not so support any role for IL-10 in the pathogenesis of ESS. This is in concordance with what has been noticed by Boelen and colleagues (1996) [15].

We observed also a highest level of IL-6 along with lowest measurements of both T3 and T4 in the MI patients while the least change was noticed in patients with chronic illness exemplified by CHF. This is in accordance with the hypothesis that the magnitude of TH alteration parallels the severity of the associated NTI [40-42]. Similarly, the considerably increased IL-10 in our MI patients was found to be linked, at least statistically, to the detected ESS. Such increase in IL-10 was reported to be beneficial to ICU patients through improving their outcome [43]. In fact, Kimur and co-workers (2001) have correlated the increased IL-6 and IL-10 with TH alterations in their 20 acute MI patients and the time course of both interleukins and T3 seemed to be tightly linked [44].

The least disturbance in T3, T4, and TSH levels concomitant with the lowest levels of IL-6 and IL-10 were observed in CHF patients, yet higher than control subjects. This is more or less similar to the findings of Nishino and associates (2000) [45] as well as of Davis and colleagues (1996) although the latter however, have not tested IL-10 [46]. Surprising was our observation that T3 and T4 were considerably low in an appreciable proportion of CRI patients whilst their serum IL-6 (and IL-10) levels were of lesser elevations compared to other two subgroups. This might simply mean that while a probable contributory role for IL-6 is suggested, it is not the only factor involved in the pathogenesis of ESS encountered in some specific forms of NTI such as CRI. Our view is supported by a study done on 28 patients with CRF in 1994 [18] and another by Boelen and colleagues who found that only 28% of T3 levels variability was accounted for by the circulating IL-6 concentrations [11].

From our observations in chronic forms of NTI, we can suggest that an acute rather than a long-lasting perturbation of IL-6 may be involved in development of ESS. In accord with our suggestion was the work carried out by Stouthard and colleagues (1994) who tested the influence of acute compared to chronic administration of IL-6 on TH homeostasis [47]. This was further supported by Hashimoto and associates (1995) who have demonstrated an inverse association between IL-6 and TH in their paediatric patients with short-lived illness [48] but not in longer-lasting diseases [49]. The same holds true for IL-10 that seems to be only partly involved in this process [32]. The continuation of the ESS state, as has been postulated by Docter and associates (1993) [50], would however be attributed to yet unidentified factors.

## Conclusion

We can conclude that euthyroid sick syndrome occurs in many patients with a wide range of non-thyroidal illnesses in association with an appreciable perturbation in IL-6 as well as IL-10 and that its pathogenesis might be regulated by IL-6 with possible involvement of some other, yet unrecognized, key players in some specific forms of NTI as chronic renal insufficiency.

## Competing interests

The author(s) declare that they have no competing interests.

## Authors' contributions

HHAZ initiated the idea of the study, participated in its design, performed the statistical analysis of the results, participated in the coordination and drafted the manuscript. He is the corresponding author of the paper.

AAS participated in study design, performed the statistical analysis of the results, participated in the coordination and helped to draft the manuscript.

SAS conceived of the study and participated in the sequence alignment and helped to draft the manuscript.

All authors have read and approved the final version of the manuscript.

## Pre-publication history

The pre-publication history for this paper can be accessed here:


